# Improving early detection of breast cancer in sub-Saharan Africa: why mammography may not be the way forward

**DOI:** 10.1186/s12992-018-0446-6

**Published:** 2019-01-08

**Authors:** Eleanor Black, Robyn Richmond

**Affiliations:** School of Public Health & Community Medicine, University of New South Wales, Sydney, New South Wales 2052 Australia

**Keywords:** Breast cancer, Early detection, Screening, Mammography, Clinical breast examination, Breast self-examination, Sub-Saharan Africa

## Abstract

**Background and methods:**

The prevention and control of breast cancer in sub-Saharan Africa (SSA) is an increasingly critical public health issue. Breast cancer is the most frequent female cancer in SSA and mortality rates from this disease are the highest globally. Breast cancer has traditionally been considered a disease of high-income countries, and programs for early detection have been developed and implemented in these settings. However, screening programs for breast cancer in SSA have been less effective than in high-income countries. This article reviews the literature on breast cancer in SSA, focusing on early detection practices. It then examines the case for and against mammography and other early detection approaches for breast cancer in SSA.

**Results:**

Women with breast cancer in SSA are younger compared with high-income countries. Most women present with advanced disease and because treatment options are limited, have poor prognoses. Delay between symptom onset and healthcare seeking is common. Engagement with early detection practices such as mammography and breast examination is low and contributes to late stage at diagnosis.

**Discussion:**

While early detection of breast cancer through screening has contributed to important reductions in mortality in many high-income countries, most countries in SSA have not been able to implement and sustain screening programs due to financial, logistical and sociocultural constraints. Mammography is widely used in high-income countries but has several limitations in SSA and is likely to have a higher harm-to-benefit ratio. Breast self-examination and clinical breast examination are alternative early detection methods which are more widely used by women in SSA compared with mammography, and are less resource intensive. An alternative approach to breast cancer screening programs for SSA is clinical downstaging, where the focus is on detecting breast cancer earlier in symptomatic women. Evidence demonstrates effectiveness of clinical downstaging among women presenting with late stage disease.

**Conclusions:**

Approaches for early detection of breast cancer in SSA need to be context-specific. While screening programs with mammography have been effective in high-income countries, evidence suggests that other strategies might be equally important in reducing mortality from breast cancer, particularly in low-resource settings. There is a strong argument for further research into the feasability and acceptability of clinical downstaging for the control of breast cancer in SSA.

**Electronic supplementary material:**

The online version of this article (10.1186/s12992-018-0446-6) contains supplementary material, which is available to authorized users.

## Background and methods

Breast cancer is a significant public health issue in sub-Saharan Africa (SSA), and the burden caused by this disease is projected to worsen [1]. While breast cancer incidence is currently lower in SSA compared with high-income countries, mortality rates are disproportionately high, and incidence is increasing [2, 3]. This has partly been driven by the epidemiological transition occurring in SSA due to an ageing population, improved control of infectious diseases, and increased prevalence of risk factors for noncommunicable diseases associated with urbanisation and economic development [1, 4]. In addition, late stage at diagnosis coupled with a paucity of comprehensive treatment centres leads to poor prognoses for women with breast cancer in SSA and contributes to high mortality rates from this disease [5, 6]. Despite the growing burden of breast cancer in SSA, it receives relatively low public health priority [4].

In this article we examine the case for and against mammography and other early detection approaches for breast cancer in SSA and suggest ways to improve early detection and work towards reducing breast cancer mortality in this region. This is based on a comprehensive review of the literature evaluating the current burden of breast cancer in SSA, associated risk factors, clinical features, and engagement with early detection practices. Literature on the effectiveness of different early detection measures in high-income and low-income settings was also reviewed. Articles were identified through searching and reviewing published literature as well as data from the International Agency for Research on Cancer (IARC). Medline, PubMed, Embase, Scopus, and Clinahl databases were searched using MeSH search terms including ‘breast cancer’, ‘screening’, ‘early detection’, ‘sub-Saharan Africa’, and ‘low-income countries’. Articles that focused on the epidemiology, clinical features, and early detection practices were included. The article aims to present an evidence-based, balanced approach to guide improvements in the early detection of breast cancer in SSA. It does this through a context-specific lens.

### Breast cancer burden in SSA

Breast cancer is the most common female cancer globally, with an estimated 1.67 million incident cases in 2012 [7]. The highest age-standardised incidence rates are observed in North America, Australia, New Zealand, and Northern and Western Europe [7] and there is an observed correlation between GDP and incidence rates [8]. However, while incidence has plateaued and is decreasing in many high-income countries, it continues to increase in low- and middle-income countries (LMICs) due in large part to the epidemiological transition described earlier [5, 8, 9]. Whereas breast cancer mortality in many high-income countries has decreased significantly over the past 25 years due to improvements in awareness, early detection and treatments [5], it is now the leading cause of death from cancer in LMICs [10]. The mortality-to-incidence ratio is higher among countries with lower GDPs [2], and the gap between high and low-income countries with respect to breast cancer mortality is expected to widen if current epidemiological trends continue [1].

Breast cancer is the most frequent female cancer among women in SSA, with 94,378 cases in 2012 (compared with 93,225 cases of cervical cancer, the second most frequent female cancer) [7, 11]. Globally, mortality from breast cancer is highest in SSA, with a five-year survival rate of less than 40%, compared with 86% in the United States [12]. Because most SSA countries lack national cancer registries, the true burden and incidence of cancer in SSA is poorly understood and probably under-reported [13]. Population-based cancer registries cover only 11% of the SSA population, and those considered ‘high-quality’ and included in analyses by the IARC only cover 2% of the total population [7]. The most recent IARC data indicates that in 2012 the total number of incident cases in SSA was 94,378 and that there were 47,583 deaths [7]. Regionally, higher age-standardised incidence rates are observed in Southern and Western Africa, and mortality is highest in Western Africa. Prevalence is lower in Southern and Central Africa compared with Eastern and Western regions [4, 7] (see Table 1).

**Table 1:** Estimated number of incident cases, number of deaths, and number of 1-year prevalent cases among women in sub-Saharan Africa, by region, 2012

	Age-standardised incidence (per 100,000 women)	Age-standardised mortality (per 100,000 women)	Number of prevalent cases over one year (thousands)
Central Africa	26.8	14.9	8.9
Eastern Africa	30.4	15.6	27.0
Western Africa	38.6	20.1	33.3
Southern Africa	38.9	15.5	8.4

Source: Ferlay J, Soerjomataram I, Ervik M, Dikshit R, Eser S, Maters C, Rebelo M, Parkin DM, Forman D, Bray F (2013) GLOBOCAN 2012 v1.0, Cancer Incidence and Mortality Worlwide: IARC CancerBase No. 11 [Internet]. Lyon, France. International Agency for Research on Cancer. Available from: http://globocan.iarc.fr, accessed on 1st April 2018

See Additional file 1 for regional classification of SSA countries

Limited published data on temporal trends demonstrates that the incidence of breast cancer in SSA is increasing. For example, data from the Zimbabwe National Cancer Registry indicates that the age-standardised incidence rate of breast cancer among women in Zimbabwe more than doubled from 20.9 in the period 1991–1995 to 46.8 in the period 2006–2010, with an average annual percentage change of 4.9% [14]. Data from the Kampala Cancer Registry in Uganda demonstrates an increase in the age-standardised incidence rate per 100,000 women from 19.4 in 1993 to 31.7 in 2007 [7].

### Risk factors

Established risk factors for breast cancer include reproductive and hormonal factors (early menarche; late menopause; nulliparity; exogenous oestrogens), genetics, age, adiposity, alcohol consumption, and low socioeconomic status [10]. Research has indicated that risk factors for breast cancer in SSA are the same as those affecting women in high-income countries [13], although there is ongoing investigation into whether additional factors, such as differences in biological characteristics of tumours, contribute to the younger age at diagnosis observed among women in SSA. Studies on the role of risk factors unique to SSA, including infections and environmental exposures, are limited and not yet conclusive. However, this is an important area of research with some interesting hypotheses that are yet to be excluded. For example, whether there is an association between insecticide exposure (common in malaria-endemic regions) and hormone receptor positive breast cancers in SSA [15].

### Clinical features

Women with breast cancer in SSA present at younger ages compared with high-income countries. Data from population-based registries and community-based studies indicate that 70% of women with breast cancer in SSA are aged 50 or less [3], and that age at diagnosis is on average 10 years younger than women in high-income countries [15, 16]. Sighoko and colleagues [3] analysed data accumulated over 23 years of population-based cancer registration in Mali and reported that the greatest increase in breast cancer incidence during this period was observed among women aged 50 and under, while a similar trend towards younger age at diagnosis was not observed for other cancers analysed over the same time period, such as cervical and stomach cancer. A widely accepted explanation for the younger age at diagnosis has been the younger population age structure in SSA, but Sighoko and colleagues’ findings [3] demonstrate that this is not conclusive. Further investigation into whether additional factors are responsible for these differences is ongoing.

Up to 80% of women with breast cancer in SSA are diagnosed with late stage (stage III or IV) disease, compared with 15% of women in high-income countries [13]. Stage at diagnosis is an important determinant of survival rates, with improved prognosis observed among women diagnosed at earlier stages of breast cancer [9]. The high rate of late stage disease in SSA has been attributed to patient- and provider-related factors. Patient-related factors include low awareness of breast cancer and the importance of early detection. Provider-related factors include limited early detection programs, and an absence of facilities for diagnosis [9].

The role of sociocultural factors in delaying early detection practices and consultation with healthcare services has also been widely reported [6, 15, 16]. Fatalism related to a breast cancer diagnosis is common among women in SSA, and indeed understandable, as many women know of somebody who has died from the disease [6, 15]. There are also cultural beliefs and stigma related to breast cancer treatments that hinder health-seeking and early diagnosis. For example, in some regions supernatural forces are though to cause breast cancer, resulting in a diminished sense of personal control over its outcome. In many countries, the ideal of beauty involves a ‘whole’ woman, so that the possibility of needing a mastectomy may provoke a sense of worthlessness for some women and deter early health seeking behaviour [16].

### Treatment

Treatment options for advanced stages of breast cancer are limited in SSA. Most women are treated with mastectomy, partly because of limited treatment options for late stage disease, but also because of poor availability of chemotherapy and radiotherapy facilities [16, 17]. Dalal and colleagues [13] reported that around two thirds of SSA countries do not have radiotherapy facilities. Furthermore, the radiotherapy facilities that exist in SSA are estimated to meet only 18% of the projected need [15]. Facilities for histological classification and identification of tumour markers are limited, as are hormonal treatments such as tamoxifen [15].

Women undergoing diagnosis and treatment of breast cancer are often faced with prohibitive out-of-pocket expenses related to treatment as well as associated travel and accommodation costs for those not living in urban centres [15, 16]. Many women seek alternative treatments such as prayer camps and herbs. In a survey of women previously treated for breast cancer in Ghana, almost half had sought traditional therapies before seeking medical attention [17]. Adequate facilities and the capacity to offer treatment to women with breast cancer is critical to reducing mortality, and should be a focus of public health efforts in SSA.

## Results

### Disease stage at diagnosis

In this review, 32 studies were identified that reported the stage at diagnosis of histologically proven breast cancer in SSA countries (summarised in Table 1). Most of these were from Western Africa, while there were fewer studies available from Central Africa. Some countries had no data available, while others (notably South Africa and Nigeria) were over-represented. The majority were retrospective studies of medical records with sample sizes ranging from 34 to 1361. Advanced stage (III or IV) at diagnosis was extremely high and ranged from 42.2% of cases among women attending a tertiary hospital in South Africa, to 98% of women attending a medical centre in Tanzania. In one South African study that compared disease stage between black and white women, disease was more advanced among black women [18]. A Nigeria study reported a mean delay of 11.2 months between symptom onset and attendance at a healthcare facility, and 80% of women in the study had advanced stage disease [19] (see Table 2). Delay between symptom onset and medical consultation is common among women in SSA, and is frequently greater than 6 months [6, 20]. This suggests that improving early detection of breast cancer among women in SSA could have a considerable impact on stage at diagnosis, and confer improved prognoses and treatment options.

**Table 2:** Literature review of stage at diagnosis of histologically proven breast cancer in SSA, by region

Year	Author	Country	Population	Sample Size	Prevalence (%)				Advanced Disease (%)
					Stage I	Stage II	Stage III	Stage IV	
*West Africa*									
2008	Ukwenya et al. [33]	Nigeria	Women with BC admitted to Ahmadu Bello Teaching Hospital for definitive treatment between 2003 and 2005	111	9.0	16.2	33.3	41.4	74.7
2006	Adesunkanmi et al. [19]	Nigeria	Women with BC seen at Obafemi Awolo Teaching Hospital between 1996 and 2003 (retrospective)	212	6.0	13.2	19.3	61.3	80.6
2009	Ntekim et al. [34]	Nigeria	Women treated for BC at University College Hospital, Ibadan, Nigeria between 2003 and 2006	763	2.0	13	46	39	85.0
2010	Kene et al. [35]	Nigeria	Women with BC treated at Ahmadu Bello Teaching Hospital between 2001 and 2005 (retrospective)	103	11.7	26.2	46.6	15.5	62.1
2008	Gukas et al. [36]	Nigeria	Women with BC diagnosed at Jos University Teaching Hospital between 1999 and 2001 (retrospective)	34	0	38.2	41.2	20.6	61.8
2008	Adebamowo et al. [37]	Nigeria	Women with BC at University College Hospital between 2004 and 2006	192	4.5	9.0	38.2	48.3	86.5
1991	Ajekigbe et al. [38]	Nigeria	Women with BC presenting to Lagos University Teaching Hospital for radiotherapy between 1984 and 1989	2154	4.0	8.7	53.2	34.1	87.3
2001	Okobia et al. [39]	Nigeria	Women diagnosed with BC at the University Hospital of Benin between 1987 and 1996	77	2.6	29.9	28.9	41.6	70.5
2013	Edmund et al. [40]	Ghana	Women with BC presenting to University of Ghana Medical School between 2005 and 2009	564	16.8	32.3	26.1	24.8	50.9
2016	Gbessi et al. [41]	Benin	Women with BC presenting to the National Teaching Hospital of HKM between 2001 and 2013	109	13.7	35.8	39.5	11.0	50.5
2015	Azon-Kouanou et al. [42]	Benin	Women with BC presenting to the National Teaching Hospital of HKM of Cotonou between 2008 and 2014	63	Stage I/II 9.5		20.6	69.8	90.4
*East Africa*									
2015	Pace et al. [43]	Rwanda	Women with BC presenting to two rural referral centres in Rwanda	144	Stage I/II 24.0		52.0	24.0	76.0
2014	Sayed et al. [44]	Kenya	Women with BC diagnosed/treated at AHKUN university hospital	99	12.1	26.3	43.4	18.2	61.6
2011	Rambau et al. [45]	Tanzania	Women with BC diagnosed at Bugando Medical Center from 2002 to 2010	328	4.3	NR	64.0	NR	
2010	Burson et al. [46]	Tanzania	Women with BC attending Ocean Road Cancer Institute between 2007 and 2009	356	NR	NR	NR	NR	90.7
1997	Amir et al. [47]	Tanzania	Women with BC admitted to the Muhimbili Medical Centre in 1996	50	0.0	2.0	88.0	10.0	98.0
2012	Mabula et al. [48]	Tanzania	Women with BC admitted to Bugando Medical Centre surgical unit between 2002 and 2011	384	4.7	10.9	63.0	21.4	84.4
2013	Tesfamariam et al. [49]	Eritrea	Women with BC attending general surgical units of 3 major hospitals between 2007 and 2008	82	9.0	27.0	46.0	18.0	64.0
2008	Gakwaya et al. [50]	Uganda	Women with BC treated at Mulago Hospital between 1996 and 2000	243	3.4	19.3	51.0	26.3	77.3
2015	Galukande et al. [51]	Uganda	Women with BC treated at Mulago Hospital between 2004 and 2007 and 2010–2012	209	2.4	8.1	72.7	16.3	89.0
*Central Africa*									
2015	Lopes et al. [52]	Angola	Women with BC between 2006 and 2014 registered by the Angolan Institute of Cancer Control	1361	4.8	16.5	73.3	2.6	75.9
2015	Armando et al. [53]	Angola	Women with BC referred to the National Oncology Centre of Luanda between 2007 and 2011	163	3.2	12.4	73.8	10.6	84.2
*Southern Africa*									
2014	Dickens et al. [54]	South Africa	BC patients diagnosed at a tertiary public hospital (CHBAH) in Soweto between Oct 2006- July 2012	1071	5.0	41.0	45.7	8.3	54.0
2013	McCormack et al. [55]	South Africa	BC patients diagnosed at a tertiary public Hospital (CHBAH) in Soweto between Oct 2006 – Jan 2012 (90% of cases were black women)	1216	5.1	41.2	44.7	9.0	53.7
1985	Pegoraro et al. [18]	South Africa	Women with BC treated at the University of Natal Teaching Hospital	640	B 0.4	B 9.0	B 59.0	B 31.0	90.0
					W 27.0	W 32.0	W 34.0	W 7.0	41.0
1984	Walker et al. [56]	South Africa	Black women with BC diagnosed at Baragwanath Hospital in Soweto between 1971 and 72 and 1980–82	195	10.1	12.4	44.9	32.6	77.5
2004	Walker et al. [57]	South Africa	Black African women with BC admitted to King Edward VIII Hospital between 1994 and 1999	57	1.75	14.0	21.1	63.1	84.2
1991	Ariad et al. [58]	South Africa	Women with BC attending the Breast Clinic of the Johannesburg Hospital	58	0.0	29.4	0.0	70.6	70.6
2010	Basro et al. [59]	South Africa	Women with BC aged 35 and under registered by a tertiary hospital and private breast health centre between 2000 and 2008	139	11.4	33.4	33.3	21.9	55.2
2000	Hoffman et al. [60]	South Africa	Women with first diagnosis of BC presenting at two tertiary hospitals in Capetown	478	31.6	26.2	28.2	14.0	42.2
1993	Muguti [61]	Zimbabwe	Women with BC treated at Mpilo Central Hospital between 1987 and 1990	NR	Stage I/II 16.0		Stage III/IV 84.0		84.0

*B* Black, *W* White, *NR* Not reported

### Early detection practices

There are few studies available on the uptake of early detection and screening practices by women in SSA. Table 3 illustrates the findings from studies identified by this review that report the prevalence of breast self-examination (BSE), clinical breast examination (CBE) and mammography by women in SSA. We identified 17 studies including 8 studies from Western Africa; 5 studies from Eastern Africa; 2 studies from Central Africa; and 2 studies from Southern Africa. Lifetime prevalence of mammography was only reported by 8 of the studies and ranged from 1.6% in an urban population of Botswana to 7.8% among nurses working at a general hospital in Lagos, Nigeria. Mammography is not available in most countries of SSA [15] and is frequently only accessible by women in urban centres. Out-of-pocket costs associated with travel and accommodation for women living in semi-urban or rural settings are often prohibitive [21].

**Table 3:** Literature review of prevalence of breast cancer screening practices from community-based studies in SSA

Year	Author	Country	Population	Urban/ Rural	Sample Size	Lifetime Prevalence (%)		
						BSE	CBE	MMG
*West Africa*								
2006	Okobia et al. [62]	Nigeria	Community-dwelling women	Semi-urban	1000	43.2	9.1	NR
2009	Akhigbe et al. [63]	Nigeria	Female health workers in major public hospitals	Urban	393	77.6	NR	3.1
2005	Kayode et al. [64]	Nigeria	Female teachers in public secondary schools	Urban	341	54.8	NR	NR
2011	Isara et al. [65]	Nigeria	Female senior secondary school students	Urban	287	10.1	NR	NR
2001	Odusanya et al. [66]	Nigeria	Nurses working at a general hospital in Lagos	Urban	204	89.2	28.9	7.8
2006	Oluwatosin et al. [22]	Nigeria	Women living in Akinyele Local Government Area, Ibadan	Rural	407	6.4	2.0	NR
2012	Opoku et al. [67]	Ghana	Ghanaian women living in either Accra or Sunyani	Urban	474	32.0	12.0	2.0
2013	Sarfo et al. [68]	Ghana	Female nursing students	Urban	250	76.0	NR	NR
*East Africa*								
2013	Azage et al. [69]	Ethiopia	Female health workers in Northwest Ethiopia	Semi-urban	390	37.0	NR	NR
2014	Legesse & Gedif [25]	Ethiopia	Female household heads in Mekelle aged 20 and over	Urban	304	53.6	12.2	2.7
2014	Ameer et al. [70]	Ethiopia	Female medical students	Urban	126	23.0	NR	NR
2014	Morse et al. [71]	Tanzania	Women presenting for outpatient reproductive health care at government hospital facilities	Urban	225	33.3	6.2	NR
2010	Kiguli-Malwadde et al. [72]	Uganda	Women attending the radiology department at Mulago Hospital, Kampala	Urban	100	66.0	NR	0.0
*Central Africa*								
2012	Atanga et al. [73]	Cameroon	Women living in Buea	Urban	120	60.0	NR	NR
2015	Nde et al. [74]	Cameroon	Female university students at University of Buea in 2014	Urban	166	41.0	19.9	NR
*Southern Africa*								
2011	Tieng’O et al. [75]	Botswana	Women attending a health facility in Gaborone	Urban	375	63.5	22.7	1.6
2007	Mukupo & Mubita-Ngoma [26]	Zambia	Women aged 15–49 living in Solwezi rural district and Lusaka urban district	Rural and urban	238	5.0 urban 5.0 rural	NR	NR

*MMG* Mammography

In the studies identified by this review, BSE generally had higher participation rates compared with CBE and mammography (see Table 3). BSE lifetime prevalence rates varied greatly and ranged from 5% among women in Zambia to 89.2% among female health workers in urban Nigeria. However the prevalence of regular (monthly) BSE was much lower than lifetime prevalence across studies. Most studies identified were from urban settings. One study of over 400 rural women in Nigeria found very low lifetime prevalence for both BSE and CBE [22]. Fewer studies examined the prevalence of CBE. Among the eight studies that reported on this, lifetime prevalence was generally lower and ranged from 2% among rural women in Nigeria to 28.9% among nurses in Lagos. Women in some studies expressed a preference for CBE over BSE. For example, a study from Malawi found that many women were concerned that they might miss something when performing checks on themselves and preferred that a trained healthcare worker have this responsibility. Nearly all of the women in that study were willing to undergo CBE [23].

Studies examining attitudes and knowledge towards CBE and BSE demonstrate that improved breast cancer awareness can have significant impacts on early detection practices. For example, a cross sectional survey conducted in rural Ghana evaluated the impact of an educational program that taught women about the benefits of early detection and demonstrated BSE techniques. The study found that women who had taken part in the program were significantly more likely to practise BSE regularly [24]. An Ethiopia study found that women with higher levels of education were significantly more likely to have had CBE [25], and the women who had never had CBE mainly attributed this to not knowing that it should be performed even when asymptomatic. Other studies have found that knowledge of CBE and BSE does not necessarily translate into improved adoption of these practices, suggesting that additional factors are influential. For example, a study from Zambia comparing practices of urban and rural women found that BSE prevalence was just as low (5%) in both groups, despite knowledge about BSE being markedly better in the urban group [26].

## Discussion

### Context-specific approaches to reducing mortality

Given the high number of breast tumours detected at late stages among women in SSA, earlier detection has an important role in reducing mortality in this region [1]. In navigating a feasible approach to improving earlier detection of breast cancer, sociocultural and local health system factors must be carefully considered. There has been a tendency to apply ‘western successes’ to the problem of noncommunicable diseases in SSA – in particular, through implementation of screening programs that have been successful in controlling disease in high-income countries [2, 27]. However, simply prescribing what has worked in high-income countries, without careful consideration of its merits and likely effectiveness in the SSA context, should be avoided. The following sections consider the challenges and likely effectiveness of mammography, BSE and CBE as screening methods in SSA, and discuss the concept of ‘clinical downstaging’ as an alternative approach to screening.

### Mammography

Mammography has long been considered the gold standard for breast cancer screening in high-income countries, based on early randomised controlled trials (RCTs) that found important reductions in mortality rates in women aged 50 and over who took part in organised mammography screening programs [5]. However, more recent reviews on the effect of mammography on breast cancer mortality have produced mixed findings, and consistently found a smaller effect (in the order of a 15% reduction) among women aged below 50 [5, 8]. A recent study from Norway, where attendance for breast screening is high, found that mammography contributed only 10% to mortality reduction among women aged 50–69 years, an effect that was considerably less than expected [28]. The authors attributed the remainder of the effect to improvements in breast cancer awareness, treatment modalities, and development of other diagnostic tools [28]. Welch [29] contends that mammography has plausibly become less effective due to increased awareness of the importance of early intervention for breast cancer.

The effectiveness of mammography on mortality reduction in SSA is likely to be even lower for several reasons. Mammography screening is financially and technically challenging to implement and sustain, requiring high-quality machines, well-trained radiologists and technicians, and investments in pathology and treatment facilities [5, 8]. Mortality reductions depend on high participation rates [5] and as described earlier, participation among eligible women in SSA is low [2] (see Table 3). Furthermore, mammography is less effective for younger women due to changes in breast tissue density, and is not appropriate for detecting tumours at advanced stages [1]. As discussed earlier, the age of peak incidence for breast cancer is lower in SSA [2], and many women have advanced stage disease at the time of diagnosis.

These factors mean that the harm-to-benefit ratio from mammography is likely higher in SSA compared with high-income settings [8, 20]. False positive results from mammography are higher among younger women and lead to unnecessary patient angst and further testing, with its inherent costs [5]. Over-diagnosis, the detection by screening of histologically confirmed breast cancers that would have otherwise remained sub-clinical, is more common among younger women and may result in unnecessary treatment [8, 29]. The harm-to-benefit ratio of a screening program is higher in populations with lower incidence, as is the case in SSA, because more women are exposed to radiation and false positive results, and fewer women receive benefit [2].

### Breast self-examination (BSE)

Although BSE was the most commonly practised early detection technique in most of the studies in this review, evidence for its effectiveness is disappointing. A Cochrane Review of two large RCTs (in Shanghai and in Russia) found no beneficial effect from BSE screening compared with no intervention, and there were twice as many biopsies of benign lesions in the BSE group [8]. Based on data from RCTs and observational studies, the IARC has reported that BSE does not seem to have a significant effect on mortality reduction for breast cancer [10], and the World Health Organization recommends that BSE should not be promoted on a population-wide level [8]. Over-detection of benign lumps can lead to unnecessary physician visits and diagnosis-related expenses, which are undesirable in the SSA setting where health facilities and resources are limited. However, Corbex and colleagues [8] note that the Russia and Shanghai RCT study populations had relatively early clinical stages at diagnosis, and suggest that greater mortality reduction from BSE might be found in populations where later stage disease at diagnosis is more common. While current evidence does not support BSE as a screening approach for breast cancer, teaching BSE at the individual level in countries where most women present with late stage disease could improve awareness of breast cancer and lead to earlier stage at diagnosis.

### Clinical breast examination (CBE)

CBE has the advantages of being a relatively simple and inexpensive technique for early detection of breast tumours [10]. Studies from LMICs have shown that it can adequately be performed by trained, non-medical health workers [8]. An ongoing RCT in Mumbai demonstrated that women who received CBE had earlier detection of breast cancers, which were significantly smaller and less advanced than their counterparts who received no screening. Results on mortality are yet to be reported [30]. A cost-effectiveness analysis of breast cancer control strategies in Ghana found that biennial CBE (combined with treatment) was the most cost-effective intervention, and the incremental cost per disability-adjusted life years saved was around 10 times lower than mammography screening [31].

Women in some of the studies included in this review had a preference for CBE over BSE, having more trust in healthcare workers than themselves in detecting an abnormality. The contact with a healthcare worker that is necessary for CBE is arguably an important advantage of this technique, as women with clinical abnormalities can be linked to health care at the time that the abnormality is detected. This would conceivably minimise time to diagnosis and treatment and reduce the loss to follow-up that too frequently occurs before initiation of treatment. However, despite the promise of CBE for early detection in SSA, implementing this as a population-based screening program is still too resource-intensive for many countries [8, 20].

### Clinical downstaging: An alternative approach to screening

Clinical downstaging, also called ‘downwards stage migration’, describes an approach to early detection of breast cancer that centres on detecting cancers early in symptomatic women, for example those with a palpable tumour or other symptom, so that women may be diagnosed with earlier disease and an improved prognosis [20]. This approach contrasts to both population-based and opportunistic screening, which aim to detect cancer in asymptomatic women. A number of authors suggest that clinical downstaging is the preferred alternative for early detection in settings where women present with late stage disease [8, 20]. There is little research on the effectiveness of downstaging, but existing research indicates it is effective in settings where most women have advanced disease. For example, a program in Malaysia that involved training health staff in CBE and increasing public awareness of symptoms of breast cancer led to a decrease in late stage disease from 60 to 35% over 4 years [32]. Studies in Sudan and Tanzania have demonstrated that CBE combined with awareness campaigns and training of health workers can be effective in downstaging breast cancer [20].

Ideally, clinical downstaging involves education of health workers and the public about early symptoms of breast cancer and the benefits of early detection, along with improved referral mechanisms facilitating follow up and diagnosis with access to treatment [5, 8]. The Breast Health Global Initiative (BHGI) recommends a phased approach to implementing clinical downstaging in low-resource settings. Initial phases prepare the health system through training and development of diagnostic and treatment guidelines. Later phases focus on enhancing awareness of breast cancer among women through education and campaigns [20, 21]. Early treatment options must also be available for women if mortality reductions are to be achieved, and initiatives to improve diagnostic and treatment capability are critical to the effectiveness of downstaging [8].

## Conclusions

Renewed efforts and resources are needed to address the burden from breast cancer in SSA, where incidence rates are increasing steadily and mortality rates are high. The combination of late stage at diagnosis and limited treatment facilities combine to severely limit a woman’s hope of full recovery from breast cancer. Despite the scale of the problem, studies on early detection are limited and some regions – especially Central Africa – are virtually unrepresented in the literature. Data on the prevalence of mammography, BSE and CBE among women in SSA indicates low engagement with early detection practices. Although mammography screening has led to mortality reduction in many high-income countries, it is currently not available in most countries of SSA, and may not be the most appropriate early detection method for this region. Positive research on the effectiveness of clinical downstaging using CBE, training of health workers, and raising public aware of breast cancer in LMICs is emerging. Clinical downstaging may be a more appropriate and effective approach to breast cancer mortality reduction in SSA, where resources to implement and maintain population-based screening programs are limited. Adequate diagnostic and treatment facilities are also required for clinical downstaging to effectively reduce mortality.

## Additional file

Additional file 1: Regions of sub-Saharan Africa. A list of sub-Saharan African countries grouped by geographical region. (DOCX 129 kb)
