# Supplementary material for: Improving early detection of breast cancer in sub-Saharan Africa: why mammography may not be the way forward
Source: Global Health. 2019 Jan 8;15:3. doi: 10.1186/s12992-018-0446-6 (PMC6325810; doi:10.1186/s12992-018-0446-6)
Supplement: Supplementary file 1 — Regions of sub-Saharan Africa. A list of sub-Saharan African countries grouped by geographical region. (DOCX 129 kb) [file 12992_2018_446_MOESM1_ESM.docx]

Appendix A. Regions of sub-Saharan Africa

| **Eastern Africa**  Burundi  Comoros  Djibouti  Eritrea  Ethiopia  Kenya  Madagascar  Malawi  Mauritius  Mozambique  Rwanda  Seychelles  Somalia  South Sudan  Tanzania  Uganda  Zambia  Zimbabwe |
| --- |
| **Central Africa**  Angola  Cameroon  Chad  Congo  DR Congo  Equatorial Guinea  Gabon  S. Tome & Prin. |
| **Western Africa**  Benin  Burkina Faso  Cote d’Ivoire  Gambia  Ghana  Guinea  Guinea-Bissau  Liberia  Mali  Mauritania  Niger  Nigeria  Senegal  Sierra Leone |
| **Southern Africa**  Botswana Lesotho  Namibia  South Africa  Swaziland |
